# Bioethics of Clinical Applications of Stem Cells

**DOI:** 10.3390/ijms18040814

**Published:** 2017-04-12

**Authors:** Carlo Petrini

**Affiliations:** Bioethics Unit, Office of the President, National Institute of Health, Via Giano della Bella 34, I-00162 Roma, Italy; carlo.petrini@iss.it; Tel.: +39-06-4990-4021 or +39-06-9787-4021; Fax: +39-06-4990-4303

**Keywords:** bioethics, clinical ethics, stem cells

## Abstract

The clinical applications of stem cells pose a multitude of problems, including safety, efficacy, information and consent, the right to unproven treatments, the “right to try”, costs, access, sustainability, scientific scrupulousness, patents and regulatory aspects, to name but a few. This article does not address individual issues, but rather introduces and discusses some of the possible approaches to solving the problems. The first part compares the consequentialist and deontological approaches, offering an overview of “top–down” and “bottom–up” models and proposing the principles of personalism as applied in clinical settings. The second part of the article suggests practical frameworks for organising the ethical issues, focusing in particular on the medical indications, patient preferences, quality of life, and contextual features.

## 1. Introduction

I am going to start by discussing a runaway trolley rushing downhill with broken brakes.

Some may be surprised at this choice of topic, but there is a reason. The subject I have been assigned, “Bioethics of clinical applications”, covers a truly vast area. A merely partial list of the ethical problems involved in the clinical application of stem cells might include: safety, efficacy, information and consent, the right to unproven treatments, the “right to try”, costs, access, sustainability, scientific scrupulousness, patents, regulatory aspects, and lots more, and still not be exhaustive. As it is not possible to address each of these topics separately, any decision to select one or more of those on the list would be arbitrary, so I propose instead to start from a totally different angle. Rather than attempting to address specific problems, I shall attempt to propose some considerations on the possible methods for addressing the problems. Hence the runaway trolley.

I should like to banish all doubt. I have taken the trolley car purely as a theoretic model for the criteria on which to base options for action: the issue we are dealing with here concerns the care of patients, which is a radically different situation. The example is merely a thought experiment, first introduced by Philippa Foot [1,2]. It also has a sobriquet: “trolleyology”.

The second part of the report nonetheless includes practical and concrete proposals for criteria regarding the points listed.

## 2. Trolleyology: Utilitarianism vs. Consequentialism

Imagine the situation: the trolley is gathering speed as it rushes down the track towards a point where it forks into two branches. On one branch, five men are working; on the other, only one. The points are set to send the trolley car to the branch where the five men are working. Obviously, all five will be crushed to death. Now imagine you are a man watching this scene and you realise that near the track there is a lever that can switch the points. If you pull the lever the trolley will be diverted onto the other track, where one man is at work. One man would be killed, but five would be saved. The question is: would you pull the lever? Many, or perhaps most, would probably answer “Yes, I would pull the lever”, in other words, you would divert the trolley onto the other track and save five lives. If, however, we also ask “Why?” the answer becomes more difficult. On what basis do we decide which is the right thing to do?

Anyone who answers that they would divert the course of the trolley is applying a consequentialist model of morals. Consequentialists [3] judge actions on the basis of their consequences, and choose whichever option causes the least harm (or, if possible, the most benefit). Consequentialism is a form of utilitarianism [4], the approach advocated notably by Bentham [5] and Mill [6], who affirmed that whatever brings the most benefit and least harm to the greatest number of people is right.

However, some people might instead reply: “No, the trolley should not be diverted”, their argument being that while it is true that if it is not diverted the trolley will kill five people rather than one, by refraining from action they would have no direct responsibility. Diverting the trolley would instead be a deliberate act and a violation of moral deontology. Deontologism [7] judges the morality of acts on the basis of their intrinsic nature, regardless of their consequences: it is based on precepts such as “thou shalt not kill”, “thou shalt not bear false witness”, etc. This is the Kantian [8] perspective, in which morality is based on categorical imperatives [9].

Now try to imagine a slightly different situation: the runaway trolley is still there with its broken brakes and the five men are still working on the track. You, however, are standing on a bridge overlooking the track. If you were to throw yourself off the bridge into the path of the trolley car it would simply crush you and continue on its way, killing all five workers as well. However, standing next to you is a great hulk of an individual. If you were to push him over the bridge and onto the track, his bulk would block the trolley and the five workers would be saved. Would you push him [10]? Most of you would certainly say “No, I wouldn’t”. This is where the utilitarian theory begins to vacillate. The result of your pushing the large man would be identical to that of pulling the lever to divert the trolley: one person would die, but five would be saved. In this case, we perceive—perhaps through our emotions, rather than through our reasoning—that to push the large man is an act we should not perform. I shall return later to the difference between pulling the lever and pushing the large man.

The trolley problem is dramatically topical. It is a dilemma posed by the algorithms that govern driverless cars. Imagine for a moment that a fast-moving driverless car suddenly detects a group of pedestrians in front of it. If the “least harm” criterion is applied then the car should swerve suddenly to avoid them, probably killing its passenger as it crashes, but saving the pedestrians [11].

## 3. A Multiplicity of Theories

Returning to the two schools of thought, consequentialism and deontologism are not the only two possible approaches to the dilemma at hand. In fact, the panorama of moral theories is far more complex, with a multitude of options to choose from: unlike the scientific method, there are many moral methods, and little agreement about which is best. To start with, both deontologism and consequentialism are “top–down” theories: each starts with a few general considerations—different for each school of thought—from which are derived the solutions for specific cases. However, there also exist “bottom–up” theories that derive their rules for conduct from specific cases [12]. Bottom–up, or inductive theories, begin with concrete particulars, rather than with abstract ideas. Inductive logic is the method used to confirm theories. If you observe enough apples falling from trees, you will conclude that apples always fall downwards, instead of upwards or sideways. You might then form a more general hypothesis that includes other falling bodies, such as pears. One of the most common inductive theories of ethics is casuistry [13], which begins by exploring the particular details of the case at hand and then reasons by finding an analogy with other similar cases. According to this theory, a decision in any particular case is more or less ethically justified depending on how well it matches the details of a prior case that is considered settled.

There are thus many other theories, including contractualism, communitarian ethics, personalism, sociobiology, subjectivism, pragmatism, liberalism, virtue ethics, new paradigm, synthetic ethics, to name but a few of the many on offer [14].

The principles associated with ontologically grounded personalism are rooted in a solid moral theory [15,16]. Where they relate to bioethics, these can be summarised as: the intangibility of human life; the therapeutic principle, whereby any intervention on life is justified only if it has a therapeutic purpose; the freedom and responsibility principle, where freedom recognises respect for life as its objective limitation; the sociality and subsidiarity principle, consisting in the achievement of the common good through individual well-being. In order to adapt these principles to the clinical fields in which the issue of stem cells and cell therapies arise, these principles could be re-formulated as: the defence of human life (every medical act should be directed towards the patient’s well-being); the principle of prevalence (the overall good of the individual takes priority over any other community good); the principle of consent (each individual must participate consciously and be free of constraint); the principle of transparency and independence.

## 4. Practical Frameworks

Some may object that all this is far too abstract and of little practical use in addressing the problems you have to solve when using stem cells in a clinical setting. At a practical level, and taking a pragmatic approach, it may help to think in terms of frameworks, which are operative and user-friendly tools that nonetheless follow a theoretic model of ethics. Frameworks are of considerable practical use, as they can be used to verify the agreement between ethical principles and practical choices. Dozens of frameworks have been proposed to help solve ethical problems in a clinical setting, and I am going to propose a modified version of one elaborated by Albert Jonsen.

Jonsen is a well-known expert in medical ethics, and his book *Clinical Ethics*, co-authored with Mark Siegler and William Winslade [17], holds that clinical dilemmas can be analysed using the following four terms of reference: (1) medical indications; (2) patient preferences; (3) quality of life; and (4) contextual features, by which are meant the social, economic, legal, and administrative contexts in which a case arises.

Jonsen’s four boxes refer to the four principles underlying the North American model of ethics known as principlism. I would not include it among the other moral theories referred to above, as it is more a method than a basic theory, and I would therefore classify it as standing half way between a moral theory and a practical framework.

Principlism and its four principles were propounded in particular in the book *Principles of Biomedical Ethics* by Tom Beauchamp and James Childress, now in its seventh edition [18]. These principles are: autonomy, beneficence, non-maleficence, and justice. The main characteristic of this model is the prima facie value of each principle: they are always valid and binding unless they are in conflict; it is not established which one takes priority over the others.

Suitably adapted, Jonsen’s framework can be used to refer to the ethical problems posed by the use of stem cells in clinical settings and orientated to the principles of personalism:

***Box 1*:** Medical indications (corresponding to the principles of beneficence and non-maleficence or, from the viewpoint of personalism, to the principle of defence of human life).

- What is the patient’s medical problem? Is the problem acute? Chronic? Critical? Reversible? Emergent? Terminal?
- What are the goals of treatment?
- In what circumstances are medical treatments not indicated?
- What are the probabilities of success of various treatment options?
- In sum, how can this patient benefit from the treatment, and how can harm be avoided?

***Box 2*:** Patient preferences (corresponding to the principle of respect for autonomy or, from the viewpoint of personalism, to the principle of freedom and responsibility).

- Has the patient been informed of benefits and risks, understood the information, and given consent?
- Is the patient mentally capable and legally competent, and is there evidence of incapacity?
- If mentally capable, what preferences about treatment is the patient stating?
- If incapacitated, has the patient expressed prior preferences?
- Who is the appropriate surrogate to make decisions for the incapacitated patient?
- Is the patient unwilling or unable to cooperate with medical treatment? If so, why?

***Box 3*:** Quality of life (corresponding to the principles of beneficence and non-maleficence and respect for autonomy or, from the viewpoint of personalism, to the principles of defence of human life and of freedom and responsibility).

- What are the prospects, with or without treatment, for a return to normal life, and what physical, mental, and social deficits might the patient experience even if treatment succeeds?
- On what grounds can anyone judge that some quality of life would be undesirable for a patient who cannot make or express such a judgment?
- Are there biases that might prejudice the provider’s evaluation of the patient’s quality of life?
- What ethical issues arise concerning improving or enhancing a patient’s quality of life?
- Do quality-of-life assessments raise any questions regarding changes in treatment plans, such as forgoing life-sustaining treatment?

***Box 4*:** Contextual features (corresponding to the principles of justice and fairness or, from the viewpoint of personalism, to the principle of sociality and subsidiarity).

- Are there professional, inter-professional, or business interests that might create conflicts of interest in the clinical treatment of patients?
- Are there parties other than clinicians and patients, such as family members, who have an interest in clinical decisions?
- What are the limits imposed on patient confidentiality by the legitimate interests of third parties?
- Are there financial factors that create conflicts of interest in clinical decisions?
- Are there problems of allocation of scarce health resources that might affect clinical decisions?
- Are there religious issues that might influence clinical decisions?
- What are the legal issues that might affect clinical decisions?
- Are there considerations of clinical research and education that might affect clinical decisions?
- Are there issues of public health and safety that affect clinical decisions?
- Are there conflicts of interest within institutions and organisations (e.g., hospitals) that may affect clinical decisions and patient welfare?

The four-box method is a useful tool for organising the relevant information. A limitation of the four-box method is that it does not help the user to resolve the problem. It merely helps clarify the question; it does not offer a solution. As shown above, the solution needs to rest on an ethical theory.

## 5. The Double Effect Principle

It is now time to return to the trolley car just to clarify the reasons for the difference between pulling the lever and pushing the large man over the bridge: in the latter case, the unfortunate person is used as a means, thereby violating the double effect principle.

The double effect principle had already entered the philosophical and ethical stage at the time of the Scholastic philosophers [19]; it has been revisited and widely debated in recent decades [20,21]. Briefly, according to this principle, an act that is performed with good intentions (such as therapy, risk prevention) but which also has harmful consequences (such as curbing freedom) is morally acceptable only if four conditions are met:

- The principal aim of the act, and the act itself, are good;
- The harmful effects are not intentionally pursued;
- The harmful effects are not the aim of the act, and the good effect is not a direct cause-and-effect result of the harmful effect;
- The intended good effect is as great as, or greater than, the harmful effects, and is proportionate to them.

In the case of the bridge, there is a direct cause and effect: the large man is used as a means, and even the exponents of utilitarianism recognise the serious moral implications of such an act.

## 6. Conclusions: Clinical Ethics in the New Era of Molecular Medicine

The bounty of molecular biology is being more and more realized in the new era of molecular medicine. This has afforded many patients access to new tailored therapies. Nevertheless, the molecular ascent of modern medicine has not been without significant consequences for the profession and medical education.

The borders of medical profession are becoming less well defined. Physicians have always been some mix of physicians and scientists, with the former the major involvement of the clinician and the latter the predominant way of life of the academic physician. Both the clinician and physician scientists shared the same vocabulary. In the last few years a new era of molecular medicine began that changed this arrangement. The new paradigm has distanced the scientists from the patient’s bedside and the clinical arena. As medical school faculties increasingly have molecular medicine as a main focal point, the clinical dialogue and the clinical ethics risk becoming emarginated [22].

In this context, sound ethical criteria in clinical medicine are particularly significant.

Clinical ethics has had a rich and complex history over the past forty years. It has been transformed from a fairly clear and straightforward set of rules and attitudes, shaped largely by the medical profession itself, into a major field of academic and social inquiry. Medical ethics taps into a wide range of specialised fields of knowledge: anthropology, economics, epidemiology, health services, research, history, law, literature, medicine, nursing, philosophy, social psychology, sociology, theology and others.

Both the moral method and the scientific method are necessary. The soundness of scientific data is crucial. To solve ethical dilemmas such as those raised by the use of stem cells in a clinical setting, reference should be made to a solid theoretical model and, if necessary, also to a practical framework. However, neither the theory nor the frameworks can relieve us of the pain of choice: the best we can hope for is to strive to improve the process by which we reach the decisions [23].

To quote Onora O’Neil in a talk she gave as part of the Gifford Lectures at Edinburgh University in 2001, “Ethical principles, like other practical principles, state abstract requirements. We cannot expect any practical principles—whether ethical or legal, social or technical—to provide a life algorithm, but the fact that principles always underdetermine action means only that they must always be complemented and implemented by the exercise of judgment” [24].
